# Direct Simulation Monte Carlo investigation of fluid characteristics and gas transport in porous microchannels

**DOI:** 10.1038/s41598-019-52707-3

**Published:** 2019-11-20

**Authors:** Vahid Shariati, Mohammad Hassan Ahmadian, Ehsan Roohi

**Affiliations:** Department of Mechanical Engineering, Faculty of Engineering, Ferdowsi University of Mashhad, P.O. Box 91775-1111, Mashhad, Iran

**Keywords:** Engineering, Mechanical engineering

## Abstract

The impetus of the current research is to use the direct simulation Monte Carlo (DSMC) algorithm to investigate fluid behaviour and gas transport in porous microchannels. Here, we demonstrate DSMC’s capability to simulate porous media up to 40% porosity. In this study, the porous geometry is generated by a random distribution of circular obstacles through the microchannel with no interpenetration between the obstacles. The influence of the morphology along with rarefaction and gas type on the apparent permeability is investigated. Moreover, the effects of porosity, solid particle’s diameter and specific surface area are considered. Our results demonstrate that although decreasing porosity intensifies tortuosity in the flow field, the tortuosity reduces at higher Knudsen numbers due to slip flow at solid boundaries. In addition, our study on two different gas species showed that the gas type affects slippage and apparent gas permeability. Finally, comparing different apparent permeability models showed that Beskok and Karniadakis model is valid only up to the early transition regime and at higher Knudsen numbers, the current data matches those models that take Knudsen diffusion into account as well.

## Introduction

Unconventional gas reservoir found its way in the world volatile energy market after severe concerns about the reduction in conventional gas output^1^. The shale rock formation that once was cynically regarded as impermeable or impractical for extraction now produces enormous quantities of natural gas. In fact, today shale gas production not only compensates the decline in conventional gas resources but for countries such as the United States has played as a turning point. Indeed, unlike previous speculations, the US is expected to be a long term supplier of natural gas even at current consumption rate^1^. Nevertheless, there is still a lingering issue, which is to accurately estimate the production prediction of unconventional reservoirs. The issue is strongly related to the morphology and flow characteristics of the media, and to address this problem, a thorough investigation should include both aspects into account.

Porous medium consists of numerous shapeless particles scattered in space, and fluid flows through interstitial volume within this media, i.e., pores. The flow is formulated by conventional Darcy’s law which in a way is analogous of Fourier’s law in heat transfer^2^. However, in microscale porous media such as shale and tight reservoirs, the pores are of the same order as mean free path, which makes the rarefaction effects become very obvious. The rarefaction can be measured by a non-dimensional parameter known as the Knudsen number (Kn). Based on this number, the flow is classified into four regimes^3^. If Kn < 0.001, the flow is in the continuum regime and since the viscous flow is the dominant transport mechanism the well-known Navier-Stokes (N-S) equations in general and Darcy’s law for porous media are applicable^4^. By increasing the Knudsen number to 0.001 < Kn < 0.1, that is, the slip regime, the non-equilibrium effect in the form of the Knudsen layer starts to appear at the solid boundaries. Then the N-S equations require to be accompanied with slip velocity boundary condition to take slippage into account as well. Even for Darcy’s law, the intrinsic permeability, recognized as characteristics of the porous structure, deviates from its value and begins to define as apparent gas permeability^5^ (*K*_*app*_). The transition regime happens when 0.1 < Kn < 10. In this situation, the intermolecular collisions are considerably less than wall collisions making the Knudsen diffusion together with slippage as the two dominant transport mechanisms. In addition, N-S equations completely fail to predict the flow behaviour^6^. In the free molecular regime, that is, Kn >10, the intermolecular collisions are negligible compared to wall collision, and Knudsen diffusion plays as the only dominant transport mechanism. For the last two regimes, i.e., Kn > 0.1, particle-based methods such as lattice Boltzmann method (LBM), direct simulation Monte Carlo (DSMC), and molecular dynamics (MD) as alternative approaches could be utilized^7^.

A grand research challenge in microscale porous media is to calculate the permeability of an unconventional reservoir, and Klinkenberg^8^ was the first who set the tone by introducing apparent gas permeability and slippage factor. Since then, a broad set of studies conducted to devise an accurate correlation for apparent permeability or slippage factor that could capture and follow the experimental results. Karniadakis and Beskok^9^ developed a Hagen-Poiseuille-type equation to obtain apparent permeability for the whole range of Knudsen numbers. Javadpour^7^ considered the flow as a combination of slippage and Knudsen diffusion effects and proposed apparent permeability as a function of pressure and temperature. Rahmanian *et al*.^10^ employed a weight factor to set the balance between viscous flow and Knudsen diffusion in way that could replicate Aguilera’s experiment. Florence *et al*.^11^ correlated slippage factor by means of experimental results, although they did not consider the effect of tortuosity. For that matter, Civan^12^ provided a more accurate correlation for Klinkenberg slippage factor as well as apparent permeability by considering the effects of porosity and tortuosity. While there are various transport models to describe apparent permeability in microscale porous media, most of them are not accurate for all gaseous regimes or have some empirical factors that must be obtained from experiments. Therefore, there are some limitations in applying them.

Recently, researchers have adopted numerical methods such as N-S, LBM, and DSMC to simulate gas flow in porous media. Moghaddam *et al*.^13^ adopted the N-S equations with the first and second-order boundary conditions to simulate gas flow in a channel and compared the results with the experimental data concluding that Karniadakis and Beskok model overestimates apparent gas permeability in the transition regime. LBM is also employed to simulate gas flow in porous media. Zhang *et al*.^14^ considered an organic capillary, showed that at higher Knudsen numbers slippage at wall boundaries enhances flow rates inside the pores. Zhao *et al*.^15^ performed a simulation in a three-dimensional digital rock by LBM. The results demonstrated that the difference between apparent permeability and intrinsic permeability increases by decreasing in pore size or pore pressure. In another attempt, Zhao *et al*.^16^ investigated the effect of heterogeneity in porous media showing that for small Knudsen number, gas mainly flows through the large pores. They also presented a more accurate proportionality factor for Klinkenberg’s model. Wang *et al*.^17^ generated porous media using quartet structure generation set (QSGS) model and studied the effect of porosity and specific surface area on the gas flow. Their outcome indicated that pore morphology plays significantly in flow behavior. A similar study by Germanous *et al*.^18^ with the QSGS model reached to the same conclusion that solid matrix complexities have a great deal of influence on both apparent and intrinsic permeabilities. DSMC, as a particle-based algorithm in rarefied gas flows, was also adopted for treating microscale porous media. Christou *et al*.^19^ simulated gas flow through nanoporous Berea sandstone by DSMC model. For ablative thermal protection system (TPS) and fuel cells, DSMC has been employed to compute permeability of these media^20^. DSMC simulation is also utilized to compute the permeability of several fibrous substrates to high-temperature gases, e.g., Jambunathan *et al*.^21^ applied CUDA-based Hybrid Approach for Octree Simulations (CHAOS) DSMC solver and obtained material properties in a pressure driven flow in Morgon felt and FiberForm TPS materials. In order to reduce the computational cost, very recently Ahmadian *et al*.^22^ implemented the dusty gas model in DSMC concluded that tortuosity has great impact on streamwise velocity, particularly at high porosity.

DSMC was originally proposed and applied to simulate high-speed transition flows^23^. In this regard, it is said that in microscale and nanoscale media with low Mach number, DSMC is not computationally efficient, and LBM as an alternative approach is more applicable^14^. It is argued that due to the probabilistic nature of the DSMC, and in order to filter any possible statistical noises happening at low Reynolds number simulation higher number of particles should be simulated as representatives of real gas molecules. However, in LBM, the number of distributed particles only depends on mesh points and lattice method^24^. Therefore, LBM requires less computational effort. Regarding that and despite the fact that DSMC still struggles with relatively high computational cost, many researchers have challenged this point of view and extended the application of DSMC by employing this approach to a broad set of microscale systems. This was accomplished with the help of certain measurements, such as new collision schemes, which addressed this issue^25^. Another reason is that the high accuracy of DSMC makes it more appealing for the researchers, i.e., even LBM is validated with the DSMC^17,26^. Moreover, while still some modifications are needed for LBM to be used for microscale porous media^27^, DSMC is capable of capturing the phenomena happening from the slip flow to free molecular regime.

The primary aim of the current research is to quantify the flow properties and permeability enhancement caused by rarefaction effects in porous microchannels over a wide range of Knudsen numbers using the DSMC method. In the present study, first through the development of a Python code, a set of solid circular particles with a specified radius were randomly distributed through the domain. Then, using the snappyHexMesh tool, the mesh generation utility of the OpenFOAM package, divided the domain into cells. Afterward, we employed dsmcFoamPlus^28^, the DSMC solver of the OpenFOAM package, to simulate rarefied gas flow in this media. The solving procedure of this solver, which is exactly the same as DSMC, will be discussed in details in the next section. Following this, the effect of critical parameters in a porous media such as porosity, particle’s diameter specific surface area and gas type on flow behavior, including velocity profile, apparent gas permeability, and mass flow rate are investigated.

## Methods

### DSMC method

DSMC is a numerical method to solve the Boltzmann equation^29,30^. It was initially proposed by Bird in 1963^31^. DSMC is a particle-based method for treating rarefied gas flow wherein every particle represents a large number of real molecules. The accuracy of the method has initiated many interests among researchers to apply to a broad range of engineering problems from spacecraft reentry to micro/nanoscale rarefied flows^32–36^.

**Figure 1:**
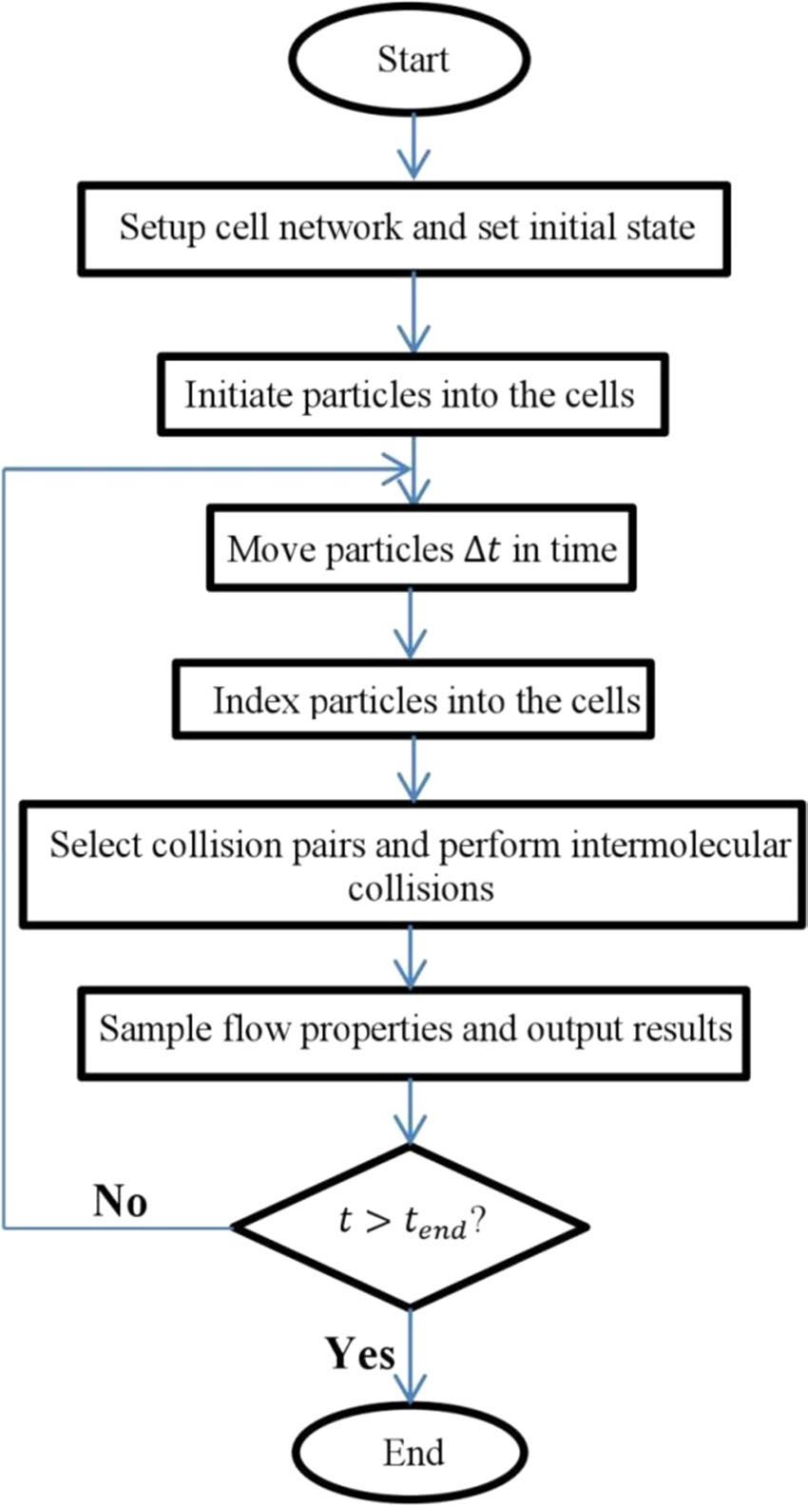
Flowchart of the DSMC method.

The primary concept of the DSMC method is to decouple the molecular movement and collision in every time step. To fulfill this purpose, the computational domain has to be divided into cells smaller than the molecular mean free path, and the simulation should be performed with a time step less than the mean collision time^37^. According to the flowchart of the DSMC presented in Fig. 1, the algorithm initiates by arranging particular number of particles in each cell. The velocity of the particles is also assigned based on the Maxwell velocity distribution function. Then, the algorithm proceeds by performing four primary steps including particle movement, indexing the particles in each cell, collision of the particles within a cell and sampling the flow properties^38^.

In every time step, each particle moves in space according to its molecular velocity. Next, each particle is indexed in its new cell according to its new position. Afterward, since in dilute gases the molecules mostly interact with each other in binary collision, pairs of particles called collision pairs are selected within each cell. Then, the probability of binary collision for collision pairs is calculated. If a binary collision is accepted, the post-collision velocities will be computed. Thereafter, by sampling from each cell, the data of the cells are collected. The sampling process involves calculating the summation of properties of particles in every cell and adding them to the total amounts in previous time steps in that cell. Finally, all thermodynamic parameters such as velocity, density, and temperature of the cells are obtained from the time-averaged sampling data over a specific period of time. These four steps are repeated until the solution time reaches the end time defined for the algorithm. For more details about DSMC algorithm, see ref.^38^.

### Intrinsic permeability

Intrinsic permeability is a parameter measuring the mobility of fluid through porous structure at the continuum regime, and it only depends on the porous geometry regardless of fluid. In the present work, to determine the intrinsic permeability, a pressure-driven flow through the porous microchannel is simulated using a finite volume solver available in the OpenFOAM package. For our simulation, we considered the flow governed by the N-S equations by^39^:

1 $$\frac{\partial }{\partial {x}_{j}}(\rho {u}_{j})=0$$

2 $$\rho {u}_{j}\frac{\partial {u}_{i}}{\partial {x}_{j}}=-\,\frac{\partial P}{\partial {x}_{i}}+\frac{{\partial }^{2}{u}_{i}}{\partial {x}_{j}\partial {x}_{j}},$$

where *ρ* is density, *μ* is the dynamic viscosity*, P* is pressure*, u*_*i*_ is the velocity in *x*_*i*_ direction (*i*, *j* = 1, 2 represents x and y direction respectively). The solver is based on the “semi-implicit method for pressure linked equations (SIMPLE)” algorithm^40^. Pressure boundary conditions are imposed at the inlet/outlet, and the solid walls have no-slip boundary conditions with the temperature held at 300 K. The intrinsic permeability for laminar flows is calculated according to Darcy’s law^41,42^:

3 $$K=-\,\frac{\mu U}{\nabla p}$$

where K is permeability of the medium, ∇*p* is the pressure gradient, *µ* is the dynamic viscosity of the fluid, and *U* is the average streamwise fluid velocity which can be measured as follows^41^:

4 $$U=\frac{1}{A}\int u\,dA,$$

wherein *u* is the streamwise velocity and *A* is total void area in a porous medium.

### Effective knudsen number

One of the important parameters in microscale media is the Knudsen number. According to this number, we find out the flow regime and all the dominant mechanisms contribute to fluid flow. In micro-porous media Knudsen number is calculated as the ratio of gas mean free path to the pore size acting as the characteristic length scale of the media. Therefore, to measure Knudsen number of a micro-porous structure first we should calculate the mean free path of the gas. Assuming the variable hard sphere (VHS) model, the mean free path of molecules is given by^43^:

5 $$\lambda =\frac{2(5-2\omega )(7-2\omega )}{15}\sqrt{\frac{m}{2\pi kT}}(\frac{\mu }{\rho }),$$

where *λ* is gas molecular mean free path, *k* is Boltzmann constant, *T* is temperature and *ω* is the viscosity-temperature index. However, in a porous medium, there is a random distribution of pore sizes. Thus, the pore size is not constant for the whole medium. In this case, the effective Knudsen number is defined as follows^41^:

6 $$K{n}^{\ast }=\frac{\lambda }{{L}^{\ast }},$$

where L* is the effective length scale of the porous medium and is determined by the following equation^44^:

7 $${L}^{\ast }=\sqrt{\frac{12{K}_{int}}{\varphi }},$$

where *K*_*int*_ is the intrinsic permeability, and *ϕ* is the porosity of the porous medium.

## Results and Discussion

The gas flow in porous media is affected by two fundamental parameters. The first parameter is the morphological characteristics, which contribute to the rarefaction as well as tortuosity of the gas flow and establishes the flow regime in the media. The second parameter is gas properties that directly influences the apparent gas permeability and determines the amount of gas production from a given reservoir. In this section, we investigate both parameters and discuss them in detail.

**Figure 2:**
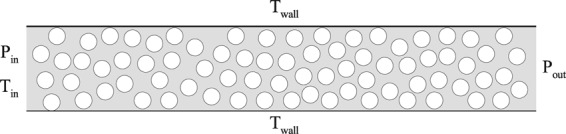
Schematic of a simulated porous geometry with the specified boundary conditions.

The simulations are performed in an *L* × *H* = 30 × 5 *μm* channel as shown in Fig. 2 with the specified boundary conditions unless otherwise mentioned throughout the paper. The porous structure is simulated as a bundle of solid particles with equal diameter. The diameter of solid particles is fixed at 1000 nm. In addition, the random distribution of solid particles in the domain is set by a python code. The channel pressure ratio, which is the ratio between inlet and outlet pressures, is set to 3 (*P*_*r*_ = *P*_*in*_/*P*_*out*_ = 3). Diffuse reflection is applied to the channel upper and lower walls along with solid particles in the domain. In addition, all wall boundaries are held at a constant temperature of 300 K, the same as the inlet temperature.

For the sake of our simulation, the computational domain is divided into 400 × 100 cells, and the time step is set to 5.0 × 10^−11^ s. The grid size and time step are set according to the DSMC setup to be less than the molecular mean free path and mean collision time, respectively. The no time counter (NTC) method^38^ is employed for intermolecular collisions. The variable hard sphere (VHS) model^45^ is applied as the collision model, and to avoid statistical noises, at least 20 molecules are set in each cell initially.

### Porous reconstruction algorithm

**Figure 3:**
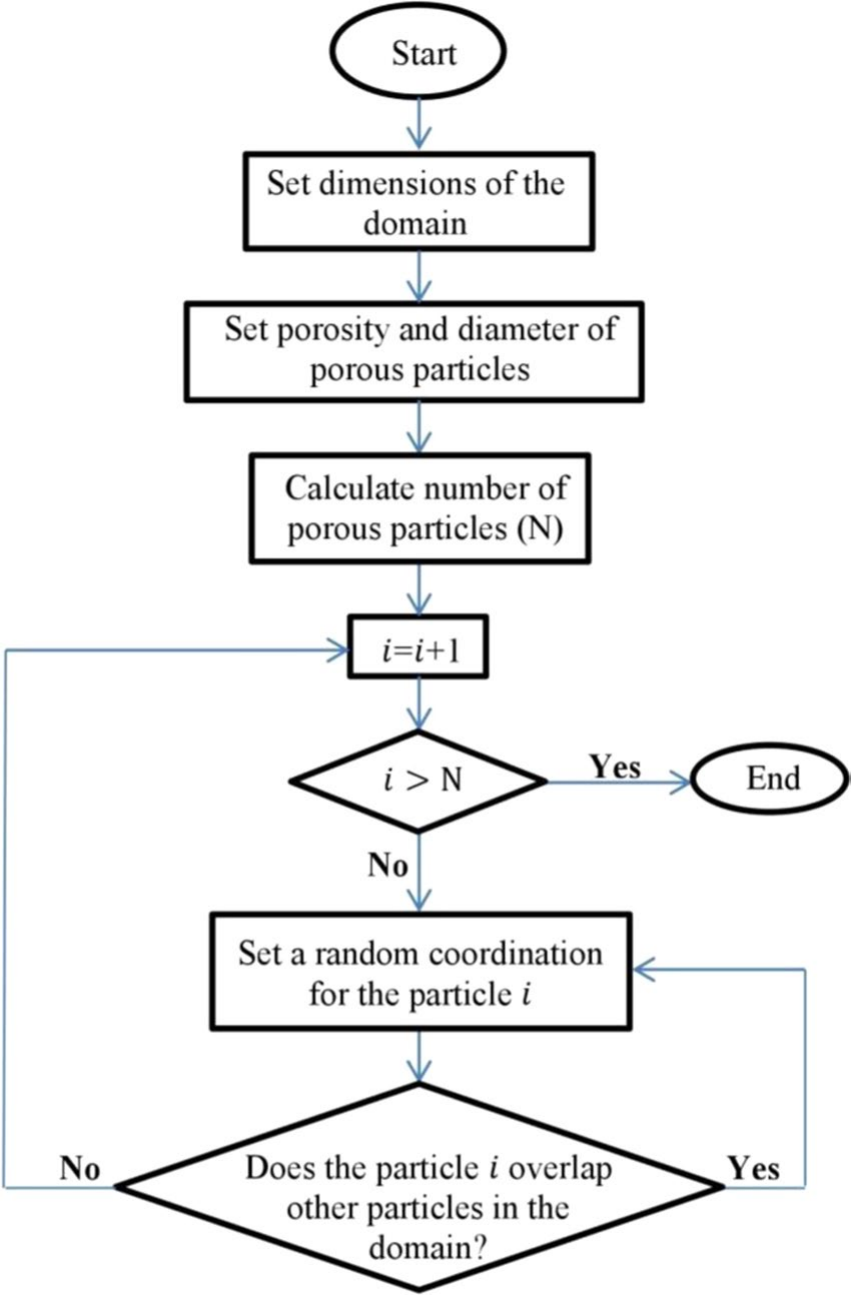
The flowchart of the algorithm used in the python code for generating porous structures.

To generate porous structures, a python code is developed to provide a random distribution of solid particles and minimize the effect of any spatial arrangement in the results. Figure 3 illustrates the flowchart of this code. Initialized by the dimensions of the domain, porosity (*ϕ*), and diameter of the solid particles (*d*_*p*_), the algorithm calculates the total area of solid particles (*A*_*solid*_) in the domain based on the definition of porosity^41^ brought in Eq. (8). In this equation *A*_*total*_ is the total area of domain.

8 $${A}_{solid}=(1-\varphi ){A}_{total}$$

Next, the algorithm obtains the total number of solid particles (*N*) according to Eq. (9) by dividing the total area of solid particles to the diameter of solid particles.

9 $$N=\frac{4{A}_{solid}}{\pi {{d}_{p}}^{2}}$$

Following that, a random function assigns a unique coordination within the domain for each particle *i* provided that the particles do not overlap each other. As a result, a random distribution of circular particles with no interpenetration forms the geometry of the porous microchannel.

### Influence of porous morphology

Porous medium comprises of abundant solid particles with various shapes which randomly distributed through the domain. This morphological complexity forms porous structure and contributes to flow patterns and transport mechanisms that happen in the media. In this section, three parameters of porosity, particle’s diameter and specific surface area as it directly relate to the structure of the media, are studied.

Porosity measures the fraction of void region of the domain^41^. In this regard, decreasing the porosity influences both morphology of porous structure, and inducing rarefaction in microscale media. To further understand the variation of this parameter, a porous microchannel with the boundary conditions stated earlier in the previous section is considered. Thereafter, the gas flow with porosities between 0.4 and 1 is simulated.

**Figure 4:**
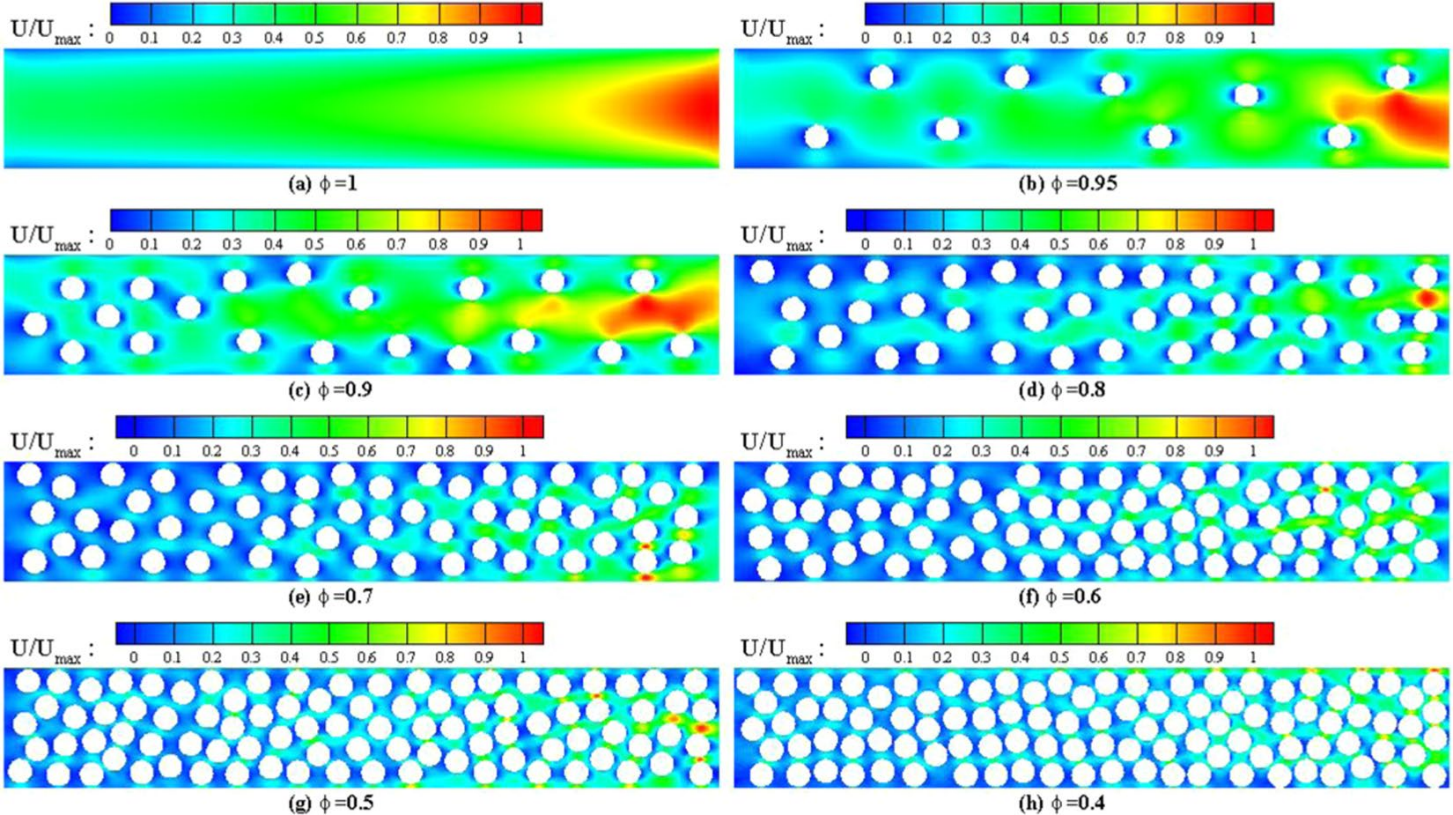
Streamwise velocity distribution of porous microchannel at different porosities. In all the cases ${{\rm{Kn}}}_{in}=0.1,{P}_{in}/{P}_{out}=3$.

Figure 4 depicts the velocity distribution of a porous microchannel with different porosities. The simulation clearly indicates that by adding solid particles; i.e., decreasing the porosity, the flow path becomes more tortuous and as a result, the flow velocity decreases. The figure also depicts that while in Fig. 4a the flow path is completely straight, adding each solid particle acts as an obstacle and the flow fluctuates around it. This pattern happens to the point that in Fig. 3h, where the domain is almost filled with solid particles, flow velocity in the whole domain considerably diminishes to the minimum magnitude.

**Figure 5:**
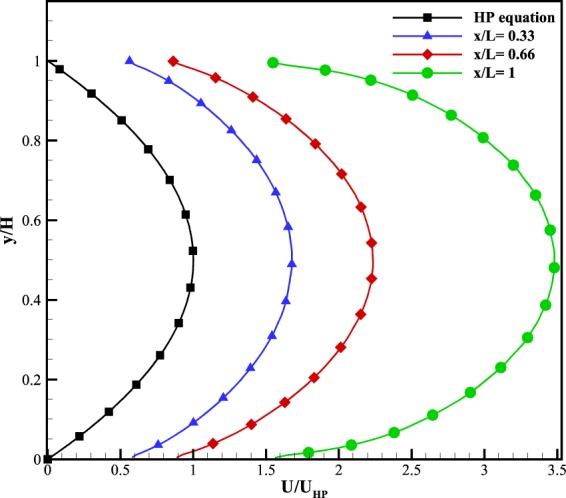
A comparison between the velocity profile of simple microchannel at three cross-sections with ${{\rm{Kn}}}_{in}=0.1,{P}_{in}/{P}_{out}=3$ and continuum Posieuille flow.

According to classical fluid mechanics, the velocity profile of a pressure-driven flow between two parallel plates in the continuum regime is given by^39^:

10 $$u=\frac{G}{2\mu }y(y-H),$$

where *u* is the streamwise velocity, *G* is the constant pressure gradient along the channel, and *H* is the height of the channel. This equation is called Hagen-Poiseuille or HP equation. To better understand the rarefied velocity profile in a microchannel and compare it to the continuum regime, Fig. 5 is plotted. This figure depicts the velocity profile of the microchannel shown in Fig. 4a at three cross-sections and compares it with the velocity profile given by the HP equation. To make a fair comparison, all the velocity profiles are normalized to the maximum velocity of the HP equation. As seen in Fig. 5, the HP equation does not depict the slippage at solid boundaries and underpredicts the maximum velocity of the channel. In addition, as the velocity increases along the channel, the velocity profile gradually loses its parabolic shape, that is, the typical velocity profile at the continuum regime. Hence, this figure illustrates that in rarefied flows not only there is slippage at solid boundaries, but also as the Knudsen number increases throughout the channel, the shape of the profile alters and loses its typical parabolic form.

**Figure 6:**
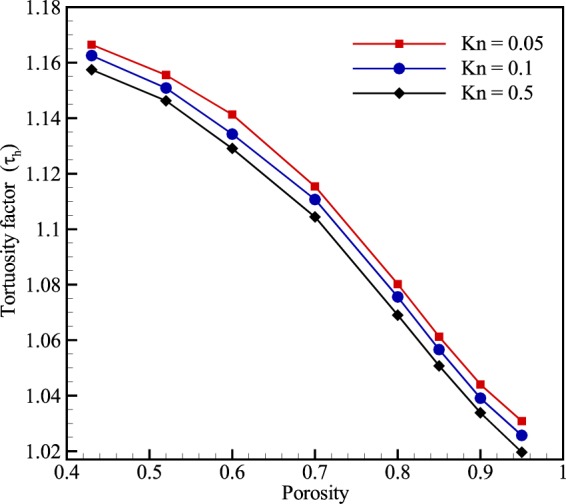
Variation of the hydraulic tortuosity (*τ*_*h*_) with porosity at different inlet Knudsen numbers.

An effective parameter in porous media is tortuosity. Tortuosity measures the average range of microscopic flow path to the length of the medium in the streamwise direction^21^. To quantify the tortuosity, the hydraulic tortuosity factor is presented as follows^18^:

11 $${T}_{i}=\frac{{l}_{t}}{l}=\frac{\langle u\rangle }{\langle {u}_{i}\rangle }$$

where 〈*u*〉 is the average velocity magnitude (average magnitude of velocity vector) of the flow in the domain and 〈*u*_*i*_〉 is the average streamwise velocity component along the pressure gradient direction within the porous media. Using Eq. (11), Fig. 6 presents the tortuosity factor of porous micro-media at different porosities and Knudsen numbers. The figure depicts that decreasing porosity makes the flow path more tortuous and also demonstrates that tortuosity decreases at higher Knudsen numbers. This means that at higher Kn number, slip velocity smooths flow path in a way that decreases the hydraulic tortuosity. The reason is that as the Knudsen number increases, the wall boundaries no longer act as a friction surface for gas particles. In contrast, the slip velocity makes the gas particles flow easier through the pore and in this way decreases the tortuosity of the flow path.

**Figure 7:**
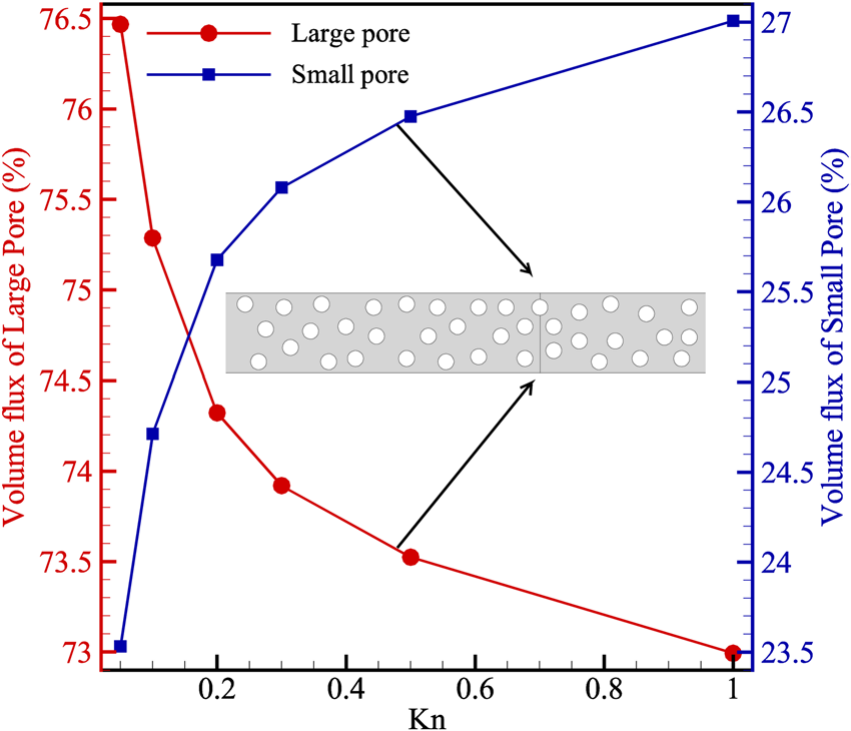
The contribution of each pore in the total volume flux passes through the cross-section.

Figure 7 depicts the volume flux passes through two pores with different pore sizes. As shown, at low Knudsen numbers, the flow tends to pass through the large pores. However, by increasing the Knudsen number, the contribution of large pores in volume flux decreases. This is because the volume flux in large pores derives by viscous flow, which is the dominant flow mechanism in continuum regime. However, when rarefaction increases, the viscous flow loses its vigour and could not carry the same volume flux through the large pores anymore. On the other hand, for the small pores, increasing the Knudsen number enhances volume flux owing to slippage at wall boundaries. Therefore, it can be concluded that the small pores permeate more volume flux at higher Knudsen numbers while the contribution of flow passage for large pores is converse.

**Figure 8:**
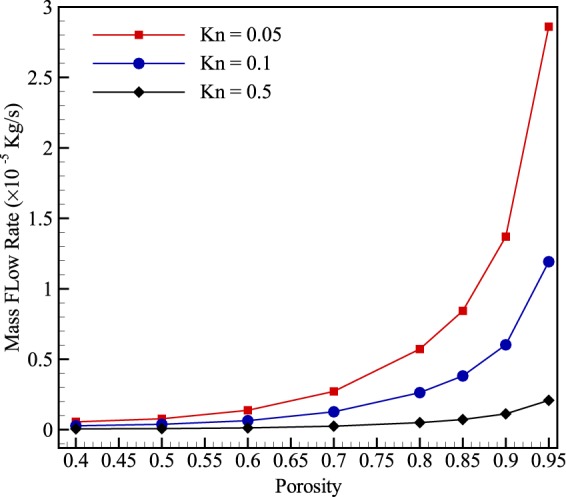
Variation of mass flow rate with porosity at different Knudsen numbers.

Figure 8 presents the variation of the mass flow rate at different porosities. As shown, reducing porosity significantly influences the mass flow rate, which is more pronounced at higher porosities. In particular, at Kn = 0.05, both slip velocity and viscous flow are the most dominant transport mechanisms and reducing porosity considerably affects viscous flow at this Knudsen number since the flow is driven by the pressure gradient. However, at higher Knudsen numbers, the dominant transport mechanism is mostly Knudsen diffusion. Hence, the molecules are not derived by the pressure gradient but by their interaction with the solid boundaries. As a result, inducing rarefaction in porous media makes the mass flow rate less altered by the structure.

**Figure 9:**
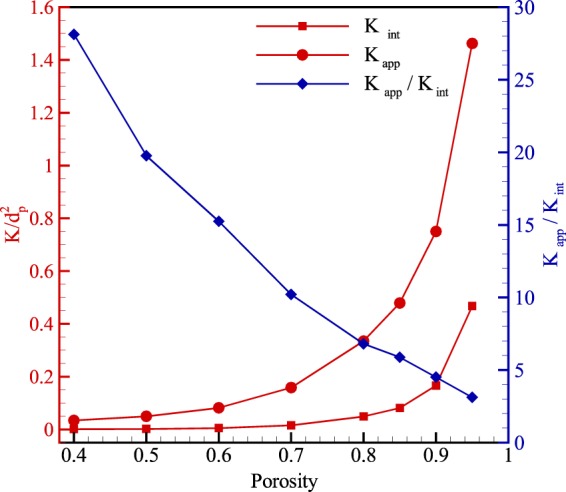
Results of intrinsic (*K*_*int*_) and apparent (*K*_*app*_) permeability normalized by the diameter of solid particles (*d*_*p*_ = 1000 *nm*) and also permeability ratio for various porosities.

Permeability, as a crucial parameter in porous media, is plotted for different porosities in Fig. 9. This figure indicates that intrinsic permeability is affected by decreasing porosity. The reason is that reducing porosity is directly related to morphology, and intrinsic permeability only depends on the morphology of porous media. The figure also indicates that intrinsic permeability is more sensitive for high porosities and is less affected by lower porosities. Furthermore, apparent permeability shows the same trend as intrinsic permeability except that the variation of apparent permeability at higher porosity is more intense than the lower one. This means that as porosity reduces, the impact of porous structures on apparent permeability becomes less significant. In addition, the increase of permeability ratio illustrates that as porosity decreases, intrinsic permeability diminishes with faster rate than apparent permeability.

**Figure 10:**
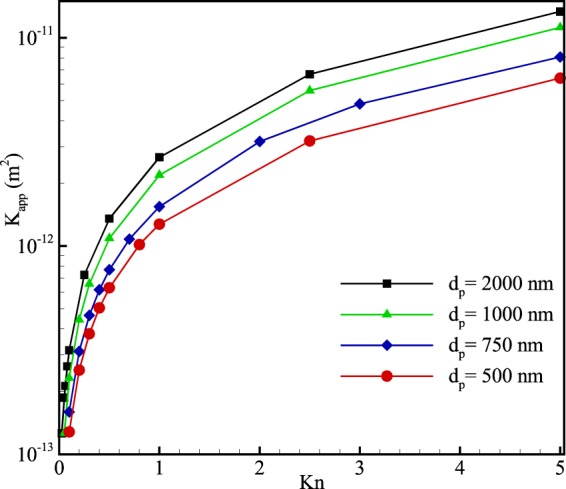
Variation of apparent permeability (*K*_*app*_) with Knudsen number for an 80% porous microchannel with different solid particle diameters.

In micro-porous media, the morphology is not bound to the porosity, whereas other factors such as the particle’s diameter can also influence the flow. In this regard, Fig. 10 presents the apparent permeability of an 80% porous microchannel with different particle diameters. According to the figure, regardless of any inlet Knudsen number, increasing particle’s diameter enhances apparent permeability of the media. To better understand this figure, the fact should be noted that increasing particle’s diameter in a constant porosity means that the flow confronts fewer but larger obstacles. Therefore, the flow experiences the same porosity concentrated in fewer porous particles. As a result, fewer porous particles make the flow path less tortuous which in turn provides less interaction with solid boundaries. Hence, it causes apparent permeability to be increased at higher particle’s diameter.

**Figure 11:**
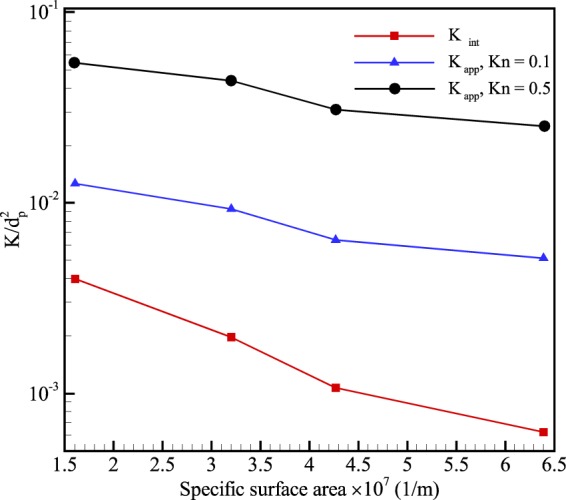
Results of permeability normalized by the diameter of solid particles (*d*_*p*_ = 1000 *nm*) at different specific surface areas.

Another morphological parameter is the specific surface area defined as the ratio of the total interstitial area to the bulk volume of the porous medium^17^. The parameter evaluates the total interface of solid-fluid in comparison to the volume of the medium. Figure 11 presents the variation of permeability with the specific surface area. Based on Eq. (3), apparent permeability (*K*_*app*_) is directly related to the average flow velocity. Thus, adding more surface area to a medium increases the interaction of solid-fluid, which means the flow encounters more friction from the boundaries. In addition, the figure shows that the influence of specific surface area diminishes at higher Knudsen numbers as the slippage weakens the impact of surface interaction. In conclusion, increasing specific surface area reduces both intrinsic and apparent permeability of the media.

### Influence of rarefaction

In micro-porous media, since the length scale of the media is of the order of mean free path the flow is in non-continuum regime^17^. However, the flow parameters are also responsible for the rarefaction in the media. In this section, pressure and temperature as the two crucial parameters influencing the mean free path are considered, and their effects are investigated.

**Figure 12:**
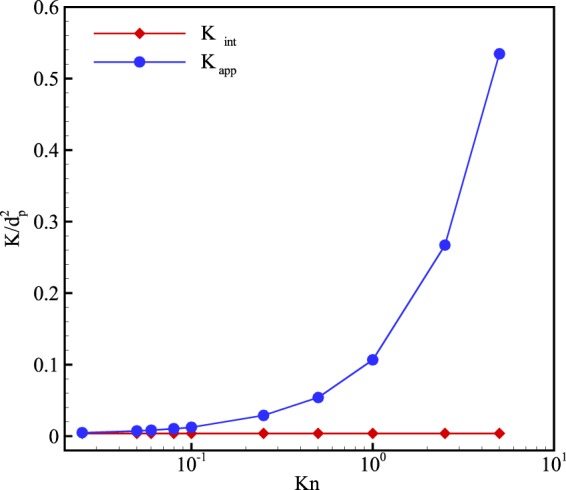
Permeability of porous media normalized by the diameter of solid particles (*d*_*p*_ = 1000 *nm*) at different inlet Knudsen numbers.

Figure 12 depicts the variation of permeability at different Knudsen numbers. To investigate the impact of the Knudsen number, the inlet pressure is decreased while the pressure ratio is held at 3. In this figure, although the intrinsic permeability (*K*_*int*_) is independent of the Knudsen number, apparent permeability (*K*_*app*_) soars extremely by decreasing the inlet pressure. Moreover, the figure shows that the apparent permeability eventually reaches intrinsic permeability at the continuum regime. This confirms the idea that apparent permeability only deviates from intrinsic permeability when the rarefaction effects appear in the flow.

**Figure 13:**
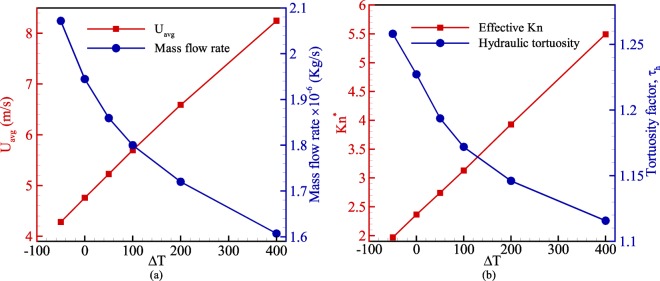
The impact of the temperature difference between inlet flow and wall boundaries on (**a**) average flow velocity and mass flow rate, (**b**) variation of effective Knudsen number and tortuosity factor.

To study the effect of temperature, a porous microchannel with 80% porosity is examined. For this purpose, the inlet temperature is set to 300 K, and the solid wall temperature is set based on temperature difference ranging from −50 to 400 K. Figure 13a presents average flow velocity and mass flow rate at different temperature differences. As depicted, applying a temperature difference to the porous medium increases flow velocity in the domain, while the mass flow rate is reduced. The reason is that the temperature difference induces rarefaction in the flow field and as a result, it strengthens slippage at solid boundaries. However, the rarefaction decreases density; therefore, the mass flow rate reduces by increasing the temperature difference. Figure 13b also indicates that as Knudsen number increases by the temperature difference in the flow, the tortuosity takes advantage of the slippage appearing at solid walls and the flow path becomes less tortuous.

**Figure 14:**
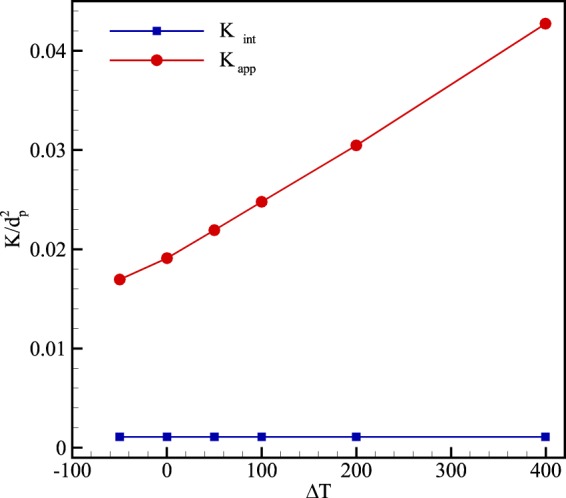
Variation of intrinsic (*K*_*int*_) and apparent (*K*_*app*_) permeability normalized by the diameter of solid particles (*d*_*p*_ = 1000 *nm*) at different temperature difference.

Figure 14 also presents apparent permeability at different temperature differences. As depicted in this figure, from Eq. (5) increasing temperature induces rarefaction in the domain. Therefore, increasing temperature differences intensifies slippage effects and accordingly enhances streamwise velocity in the flow field. In this way, apparent gas permeability which from Darcy’s law relates to the velocity of the flow increases dramatically at high pressure gradients.

### Influence of gas type

**Figure 15:**
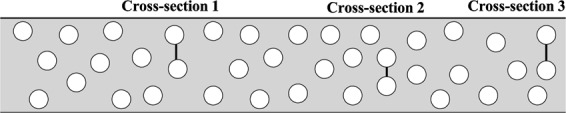
The location of three cross-sections in the 80% porosity microchannel.

In this section, we investigate the effect of gas type on a porous microchannel. For this aim, argon and methane, whose properties are reported in Table 1 are considered. The simulation is conducted for an 80% porous microchannel with the boundary conditions described in Sec. Results and discussions. For this investigation, three cross-sections as shown in Fig. 15 are considered. The location and pore size of the three cross-sections are also presented in Table 2.

**Table 1:** Properties of the gases^31^.

Gas	Symbol	Degrees of freedom (ξ)	Molecular mass (kg)	Molecular diameter (m)	Viscosity (N.s/m^2^)	Viscosity index (ω)
Argon	Ar	3	66.3 × 10^−27^	4.17 × 10^−10^	2.117 × 10^−5^	0.81
Methane	CH_4_	6.4	$26.63\times {10}^{-27}$	$4.83\times {10}^{-10}$	$1.024\times {10}^{-5}$	0.84

**Table 2:** The location and pore size of three cross-sections shown in Fig. 15.

	Cross-section 1	Cross-section 2	Cross-section 3
Location	x/L = 0.31	x/L = 0.68	x/L = 0.96
Pore size (m)	${h}_{1}=8.16e-7$	${h}_{2}=4.94e-7$	${h}_{3}=8.78e-7$

**Figure 16:**
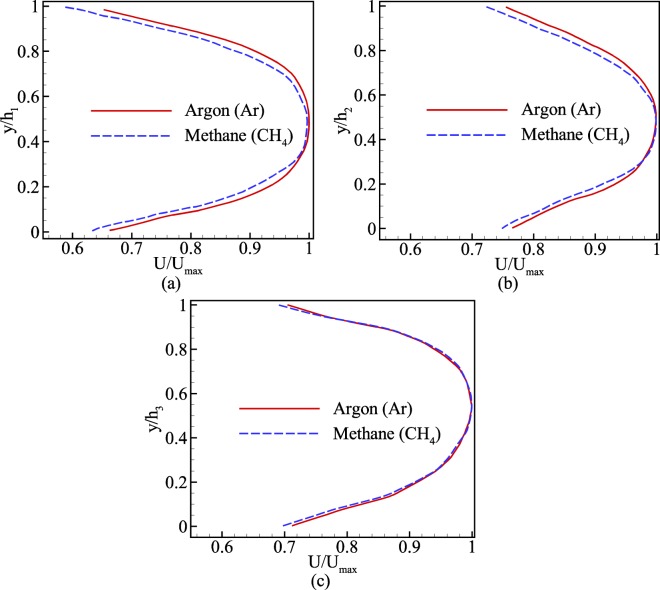
Normalized streamwise velocity profile of three cross-sections shown in Fig. 15.

Figure 16 depicts normalized velocity profile at three cross-sections shown in Fig. 15. According to this figure, it is clear that at any cross-section argon has higher slip velocity than that of methane. The reason is that according to Eq. (5) and the properties of two gases in Table 1, argon has higher molecular mass and viscosity than methane, which means that in a similar situation, the mean free path for argon is greater than that of methane. Consequently, with higher mean free path, argon experiences higher Knudsen numbers. Hence, the slip velocity for argon is higher than methane throughout the domain. In addition, a comparison among three cross-section points out that, while the Knudsen number along the channel increases, the slippage on cross-section 2 is higher than cross-sections 1 and 3. However, based on Table 2 this could be justified. Based on this table, the pore size of cross-section 2 is smaller than cross-section 1. In this regard, not only cross-section 2 has higher mean free path because of its location compared to cross-section 1, but also cross-section 2 has smaller length scale as pore size. Therefore, both mean free path and length scale provides higher Kn for cross-section 2. On the other hand, between cross-sections 1 and 3, since both have roughly the same pore size, only because cross-section 3 has lower pressure (higher mean free path), its slip velocity is higher than cross-section 1. This discrepancy among three cross-sections suggests the extensive variation of local Knudsen number in porous media.

**Figure 17:**
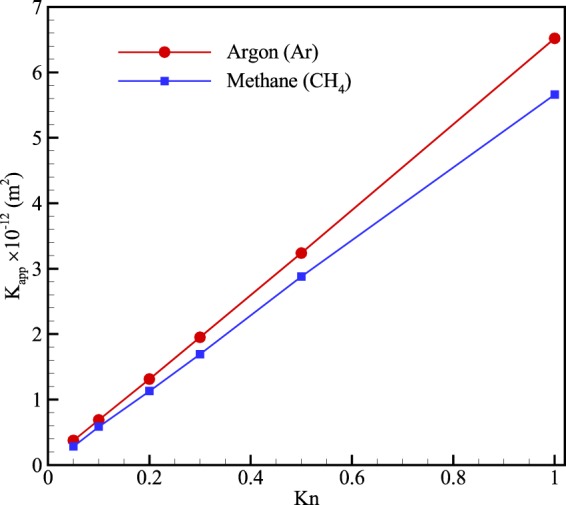
Apparent permeability (*K*_*app*_) of a porous medium for two gases at different inlet Knudsen numbers.

Figure 17 illustrates the apparent permeability for methane and argon over a wide range of inlet Knudsen numbers. According to Fig. 16, since the slip flow for argon is higher than that of methane; therefore, in a similar situation, argon has greater volume flow rate. Hence, the apparent permeability of argon must be higher than that of methane. It should also be noted that at lower Knudsen numbers, the permeability becomes less affected by the flowing gas, and consequently, the permeability of two gases reaches the same.

### Evaluation of various apparent permeability calculation models

In the past few decades, many researchers tried to propose a model to predict the permeability of porous media correctly. For this purpose, the apparent permeability models were derived by accounting morphological parameters as well as different transport mechanisms involved in this media. Based on these considerations, some formulas can be found in the literature. In this section, six apparent permeability models presented in Table 3 were selected to be compared with our DSMC results. For this comparison, we simulated a porous microchannel same as those presented in the previous sections and compared the permeability ratio against others brought in Table 3.

**Table 3:** Apparent permeability models found in the literature.

Reference	*K*_*opp*_/*K*_*int*_	Remark
Klinkenberg^8^	1 + 4*cKn*	*c* = 1.037
Beskok and Karniadakis^49^	$(1+\alpha (Kn)Kn)(1+\frac{4Kn}{1+Kn})$	$\alpha (Kn)=\frac{128}{15{\pi }^{2}}{\tan }^{-1}(4K{n}^{0.4})$
Sakhaee-Pour and Bryant^46^	$1\,+\frac{64}{3\pi }\,Kn$	Dust gas model
Mohammadmoradi and Kantzas^47^	1 + *aKn*	*a* = 9.62
Zhao *et al*.^15^	1 + 4*cKn*	*c* = 0.8
Kawagoe *et al*.^48^	$1+\frac{64}{3\pi }\frac{1+{c}_{1}^{k}p}{1+{c}_{2}^{k}p}$	${c}_{1}^{k}p=\sqrt{\frac{\pi }{2}}\frac{2}{Kn};{c}_{2}^{k}p=\sqrt{\frac{\pi }{2}}\frac{2.47}{Kn}$

**Figure 18:**
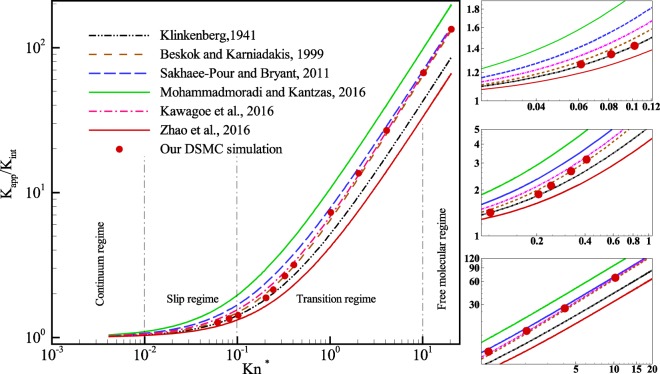
Comparison of different permeability models with the current simulation result.

Figure 18 reports predictions of the six aforementioned models along with our DSMC simulation result. Based on this figure, at low Knudsen number limited to slip flow regime, our simulation follows Klinkenberg^8^ and the B-K^9^ models since in both models the flow mechanisms are considered as viscous flow together with slippage at the solid boundaries. According to Table 3, it is observed that Sakhaee-Pour and Bryant^46^, Mohammadmoradi and Kantzas^47^, and Zhao *et al*.^15^ proposed the same Klinkenberg first order equation but with different values for the slippage factor. However, the results plotted in Fig. 15 show that they are not as accurate as Klinkenberg’s model in predicting the permeability of the media. In addition, at higher Kn number, our results deviate and gradually starts to pursue the Kawagoe’s model^48^. The reason is that in the model presented by Kawagoe *et al*. not only all three transport mechanisms, that is, viscous flow, slip velocity and Knudsen diffusion are considered, but also the effect of tortuosity was taken into account. Therefore, for the rest of the range of Kn number, our results follow the Kawagoe’s model since it has correctly formulated the flow mechanisms as well as the morphology of the porous media.

## Conclusion

In this study, the DSMC algorithm as an accurate particle-based method is adopted to simulate porous microchannels. The purpose of using DSMC method is to demonstrate its capability in solving micro-porous media. According to our findings, the method could simulate micro-porous media up to 40%, and it provides the accuracy needed to analyze the transport mechanisms involved in this media. The porous structure is assumed as a bundle of solid particles with a specified radius scattered through the domain. In addition, for the random distribution of solid particles, a Python code is developed. Then, by simulating porous media at different porosities and specific surface areas, the morphological complexity of porous structure is evaluated. Our results demonstrate that although increasing porosity intensifies tortuosity in the flow field, at higher Knudsen numbers the tortuosity reduces due to slip flow at solid boundaries. Furthermore, simulating porous media with two different gas types shows that the property of the gas itself influences the apparent permeability of media. Finally, comparing different apparent permeability models demonstrates that B-K (Beskok and Karniadakis) model and Klinkenberg’s model are only valid up to transition regime. At higher Knudsen numbers, our data matches Kawagoe’s model that takes Knudsen diffusion as well as tortuosity into account. This result demonstrates that Knudsen diffusion, which is usually neglected at slip flow regime is crucial at higher Knudsen numbers and substantially influences the apparent permeability when the flow is in the transition regime.

## Data Availability

All the data presented in this paper are available upon request.
